# Chia (*Salvia hispanica*) Gene Expression Atlas Elucidates Dynamic Spatio-Temporal Changes Associated With Plant Growth and Development

**DOI:** 10.3389/fpls.2021.667678

**Published:** 2021-07-20

**Authors:** Parul Gupta, Matthew Geniza, Sushma Naithani, Jeremy L. Phillips, Ebaad Haq, Pankaj Jaiswal

**Affiliations:** ^1^Department of Botany and Plant Pathology, Oregon State University, Corvallis, OR, United States; ^2^Molecular and Cellular Biology Graduate Program, Oregon State University, Corvallis, OR, United States

**Keywords:** Chia (*Salvia hispanica* L.), transcriptome, polyunsaturated fatty acid, superfood, expression atlas, pseudocereal grain, omega-3 fatty acids, BUSCO plant gene expression

## Abstract

Chia (*Salvia hispanica* L.), now a popular superfood and a pseudocereal, is one of the richest sources of dietary nutrients such as protein, fiber, and polyunsaturated fatty acids (PUFAs). At present, the genomic and genetic information available in the public domain for this crop are scanty, which hinders an understanding of its growth and development and genetic improvement. We report an RNA-sequencing (RNA-Seq)-based comprehensive transcriptome atlas of Chia sampled from 13 tissue types covering vegetative and reproductive growth stages. We used ~355 million high-quality reads of total ~394 million raw reads from transcriptome sequencing to generate *de novo* reference transcriptome assembly and the tissue-specific transcript assemblies. After the quality assessment of the merged assemblies and implementing redundancy reduction methods, 82,663 reference transcripts were identified. About 65,587 of 82,663 transcripts were translated into 99,307 peptides, and we were successful in assigning InterPro annotations to 45,209 peptides and gene ontology (GO) terms to 32,638 peptides. The assembled transcriptome is estimated to have the complete sequence information for ~86% of the genes found in the Chia genome. Furthermore, the analysis of 53,200 differentially expressed transcripts (DETs) revealed their distinct expression patterns in Chia's vegetative and reproductive tissues; tissue-specific networks and developmental stage-specific networks of transcription factors (TFs); and the regulation of the expression of enzyme-coding genes associated with important metabolic pathways. In addition, we identified 2,411 simple sequence repeats (SSRs) as potential genetic markers from the transcripts. Overall, this study provides a comprehensive transcriptome atlas, and SSRs, contributing to building essential genomic resources to support basic research, genome annotation, functional genomics, and molecular breeding of Chia.

## Introduction

*Salvia hispanica* L. (Chia), an annual herbaceous plant of the Lamiaceae (mint) family, is a native of Central America's highlands (Cahill, 2005; Ixtaina et al., 2008; Baginsky et al., 2016). It is a photoperiod-sensitive short-day flowering plant, which usually grows about 1 m in height and produces raceme inflorescence bearing small purple flowers. Chia, which is cultivated primarily for its seeds, is a core ingredient of the Mayan and Aztec population's diet. Recently, its consumption has grown outside South America due to its rich nutritional and gluten-free characteristics (Mohd Ali et al., 2012). Chia seeds contain ~40% oil by weight the majority of which are omega-3 and omega-6 polyunsaturated fatty acids (PUFAs) (Mohd Ali et al., 2012). The seeds are also rich in protein (15–20%), dietary fiber (20–40%), minerals (4–5%), and antioxidants (Reyes-Caudillo et al., 2008; Ayerza and Coates, 2009; Muñoz et al., 2013). These nutritional attributes have made Chia a desirable superfood and a pseudocereal. Several dietary studies in humans and mouse models suggest that a diet supplemented with Chia seeds resulted in improving muscle lipid content, cardiovascular health, total cholesterol ratio, triglyceride content (Vuksan et al., 2007, 2010, 2017a,b; Oliva et al., 2013; Valdivia-López and Tecante, 2015; Ullah et al., 2016; Marcinek and Krejpcio, 2017), and helped to attenuate blood glucose levels in patients with type-2 diabetes (Vuksan et al., 2007; Chicco et al., 2009; Peiretti and Gai, 2009; Oliva et al., 2013). Besides its food and nutrition value, Chia is a rich source of many other useful products. Its leaves contain various essential oils, such as β-caryophyllene, globulol, γ-muroleno, β-pinene, α-humulene, germacrene, and widdrol, which are known to have insect repellant or insecticidal properties (Amato et al., 2015; Elshafie et al., 2018).

High-throughput gene expression datasets from various oilseed crops, such as soybean (*Glycine max*), peanut (*Arachis hypogaea*), false flax (*Camelina sativa*) (Libault et al., 2010; Severin et al., 2010; Clevenger et al., 2016; Kagale et al., 2016), have been instrumental in understanding the key regulators of metabolic and developmental processes (Druka et al., 2006; Sekhon et al., 2011; Stelpflug et al., 2016; Cañas et al., 2017; Kudapa et al., 2018) and for translational research leading to crop improvement. Despite being a valued crop, the genomics resources available for Chia are limited. A few previous studies focusing on fatty acid metabolism have generated transcriptome data and simple sequence repeats (SSRs) from Chia seeds (Sreedhar et al., 2015; Peláez et al., 2019). Also, Wimberley et al. (2020) reported leaf and root transcriptomes and identified terpenoid biosynthesis genes. To our knowledge, an extensive plant structure and the developmental stage-specific transcriptomes for Chia have not yet been reported.

We took an initiative to build a gene expression atlas for Chia from the 13 plant structures collected from a range of vegetative and reproductive stages. Furthermore, we identified SSRs within the transcribed region of the Chia genome; added functional-structural annotations to transcripts; analyzed the differential expression of transcripts across spatial and temporal scales; and conducted a pathway enrichment analysis to understand the regulation of Chia metabolism throughout its development.

## Results

### Sequencing and *de novo* Assembly of Chia Transcriptome

**Figure 1:**
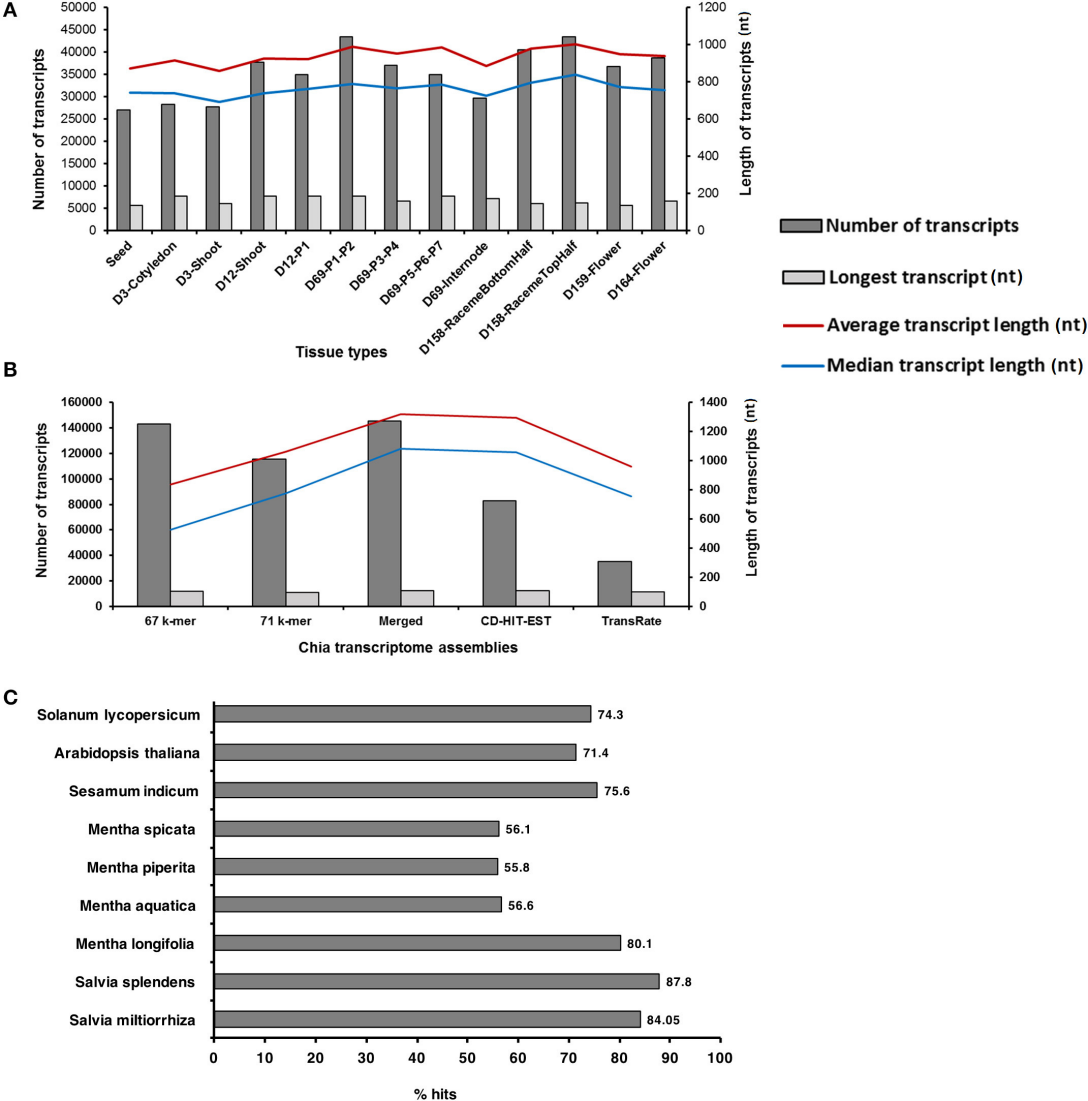
Statistics of *Salvia hispanica* transcriptome assemblies and BLAST results. **(A)** Tissue-specific assemblies; **(B)** reads from each tissue type were combined and assembled at 67 and 71 k-mer. Merged assembly of 67 and 71 k-mer, CD-HIT-EST, and TransRate assemblies by removing the redundant reads; **(C)** Comparison of *S. hispanica* transcripts with the publicly available Lamiales and eudicot gene models and peptide sets.

The transcriptome data were generated from the 13 Chia plant structures, including mature dry seeds, early seedling shoots, leaves (representing the developmental stages P1–P7), an internode between P5 and P6 leaves, top and bottom halves of pre-anthesis raceme inflorescence, and flowers from the day of anthesis [Days after flowering (DAF)], and 5 day post-anthesis (Table 1). A 101-basepair (bp) paired-end sequencing of the 39 complementary DNA (cDNA) libraries [three biological replicates for each sample prepared from the poly-A enriched messenger RNA (mRNA)] resulted in 393,645,776 sequence reads and ~80 Gb of the nucleotide sequence (Supplementary Material 1). The high-quality reads were assembled at 65 and 75 k-mer lengths, and then, after merging both k-mer assemblies for each tissue type, their unique transcripts were generated. The number of assembled transcripts ranged from 27,066 to 43,491 for tissue-specific assemblies (Figure 1A). Among the vegetative tissues, D69-P1-P2 leaf samples contained a maximum (43,491), and the seed contained a minimum (27,066) number of assembled transcripts (Figure 1A). Among reproductive structures such as inflorescence and flowers, the maximum number of transcripts (43,418) with an average length of about 1,000 bases was observed in the top half of the D158-raceme inflorescence sample (Figure 1A).

**Table 1:** Description of the plant material used for generating the Chia transcriptome atlas.

**Growth stage**	**Sample collection days after sowing (DAS)**	**Sample description**	**Sample name**
Vegetative	Day 0	Dry Seed	Seed
	Day 3	Green cotyledon	D3-Cotyledon
	Day 3	Above ground shoot parts (whole shoot)	D3-Shoot
	Day 12	Above ground shoot parts (whole shoot)	D12-Shoot
	Day 12	Very first/youngest leaf at the shoot apex	D12-P1
	Day 69	First and second leaves at the shoot apex	D69-P1-P2
	Day 69	Third and fourth leaves at the shoot apex	D69-P3-P4
	Day 69	Fifth, sixth, and seventh leaves at the shoot apex	D69-P5-P6-P7
	Day 69	Internode between the 5th and 6th leaves	D69-Internode
Reproductive	Day 158	The top half of the raceme inflorescence (pre-anthesis)	D158-RacemeTopHalf
	Day 158	The bottom half of the raceme inflorescence (with pre-anthesis flowers)	D158-RacemeBottomHalf
	Day159	Flowers from Day-1 of flowering (anthesis stage)	D159-Flowers
	Day 164	Flowers from Day-5 of flowering (anthesis stage)	D164-Flowers

Additionally, the high-quality paired-end reads (352,976,255) from all tissue libraries were pooled and assembled at 67 and 71 k-mer lengths by using Velvet (Zerbino and Birney, 2008) and Oases (Schulz et al., 2012). Chia transcript isoforms generated by each k-mer (67 and 71 k-mer lengths) assembly were merged to represent a total of 145,503 unique transcripts (≥201 bases in length) (Figure 1B). The use of the Cluster Database at High Identity with Tolerance-Expressed Sequence Tag (CD-HIT-EST) algorithm (Li and Godzik, 2006) for removing redundant transcripts (displaying ≥90% similarity) yielded 82,663 transcripts (Figure 1B). In parallel, we used a quality assessment software, the TransRate (Smith-Unna et al., 2016), on the original assembly that yielded 35,461 transcripts (Figure 1B). TransRate detects the redundant transcripts by aligning the reads to multiple transcripts and assigns all of them to the transcript that best represents a canonical form. We observed that the assembly produced by CD-HIT-EST experienced only a minor loss in the percentage of reads aligned. The assembly produced by TransRate, which utilizes Salmon (Patro et al., 2017) to estimate transcript abundance with map-based methods, contained nearly 50% fewer reads aligned in comparison to the CD-HIT-EST assembly. Furthermore, we used a quality assessment tool, QUAST (Mikheenko et al., 2016), on the original assembly and each of the redundancy reduced assemblies (Supplementary Material 2). Both original and TransRate assemblies had better statistics in transcript number and length, and also contained the worst statistics in the complementing category (Supplementary Material 2). The assembly produced by CD-HIT-EST represented the most moderate version. Based on the quality assessment and alignment statistics, we pursued the CD-HIT-EST assembly with 82,663 transcripts for downstream analyses. A workflow for an assembly and a downstream analysis is shown in Supplementary Material 3. The Benchmarking Universal Single-Copy Orthologs (BUSCO) analysis suggests that our *de novo* assembled transcriptome contains complete gene sequences for 86.4% of the Chia genes (Supplementary Material 4).

### Functional Annotation of Chia Transcriptome

We compared the 82,663 assembled Chia transcripts to the publicly available genomes and gene models of eudicots using BLASTx and tBLASTx (Mount, 2007) to estimate approximate coverage of genes represented in the assembled transcriptome. More than 84% (the number that is very close to the BUSCO analysis) of the assembled Chia transcripts were mapped to the closely related *Salvia miltiorrhiza* (Wenping et al., 2011) and *Salvia splendens* (Ge et al., 2014) transcriptomes (Figure 1C). The dispersion of coverage within the genus is not surprising since the *Salvia* genus is very diverse. Both *S. miltiorrhiza* and *S. splendens* share a common center of origin in China whereas *S. hispanica* originated in Central America. In the Lamiaceae family, about 56% of the Chia transcripts were mapped to the transcriptomes from the members of the *Mentha* genus, namely water mint (*M. aquatica*), peppermint (*M. piperita*), and spearmint (*M. spicata*) (Ahkami et al., 2015), except that 80% of the Chia transcripts were mapped to *M. longifolia* genes identified in its genome assembly (Vining et al., 2017). Moving up the taxonomic rank to the order of Lamiales, 75% of the Chia transcripts were mapped to sesame (*Sesamum indicum*) (Zhang et al., 2013), an oilseed crop. A total of 71% and 74% Chia transcripts were mapped to a proteome set of the model plants *Arabidopsis thaliana* and the *Solanum lycopersicum* (tomato), respectively (Figure 1C). Although the assembled transcriptomes were not available, two publicly available RNA-Seq reads from *S. hispanica* seeds (INSDC Accession PRJNA196477) (Sreedhar et al., 2015) and the leaf tissues (INSDC Accession PRJNA359989) (Boachon et al., 2018) were aligned against our assembled Chia transcriptome: about 69% sequence reads from the seed, and 43% from the leaf tissues were mapped to our Chia transcript assemblies.

Peptide sequences from the assembled transcripts were generated by using TransDecoder. About 65,587 of total 82,663 transcripts from Chia were translated into 99,307 peptides. The number of peptides is higher than the number of transcripts assembled due to the occurrence of multiple open reading frames (ORFs) in a single transcript. Functional annotation of peptides was first carried out by using InterProScan (Jones et al., 2014) to assign structural-functional domains and then carried out by employing the agriGO ontology enrichment analysis (Tian et al., 2017). We were successful in assigning InterPro accessions to the 45,209 peptides (Supplementary Material 5), and gene ontology (GO) terms to a total of 32,638 peptides (Supplementary Material 6). A total of 20,857 peptides were annotated with GO biological process (BP); 8,677 peptides were annotated with GO cellular component (CC) terms, and 26,877 peptides were annotated to GO molecular function (MF) terms (Supplementary Material 6).

### The Vegetative and Reproductive Plant Structures of Chia Show Distinct Gene Expression Patterns

The RNA-Seq by expectation-maximization (RSEM) package (Li and Dewey, 2011) was used to calculate Fragments Per Kilobase of transcript per Million mapped reads (FPKM) value for the final set of 82,663 assembled transcripts from Chia. After removing the transcripts with an extremely low expression, we considered 82,385 transcripts for further analysis.

**Figure 2:**
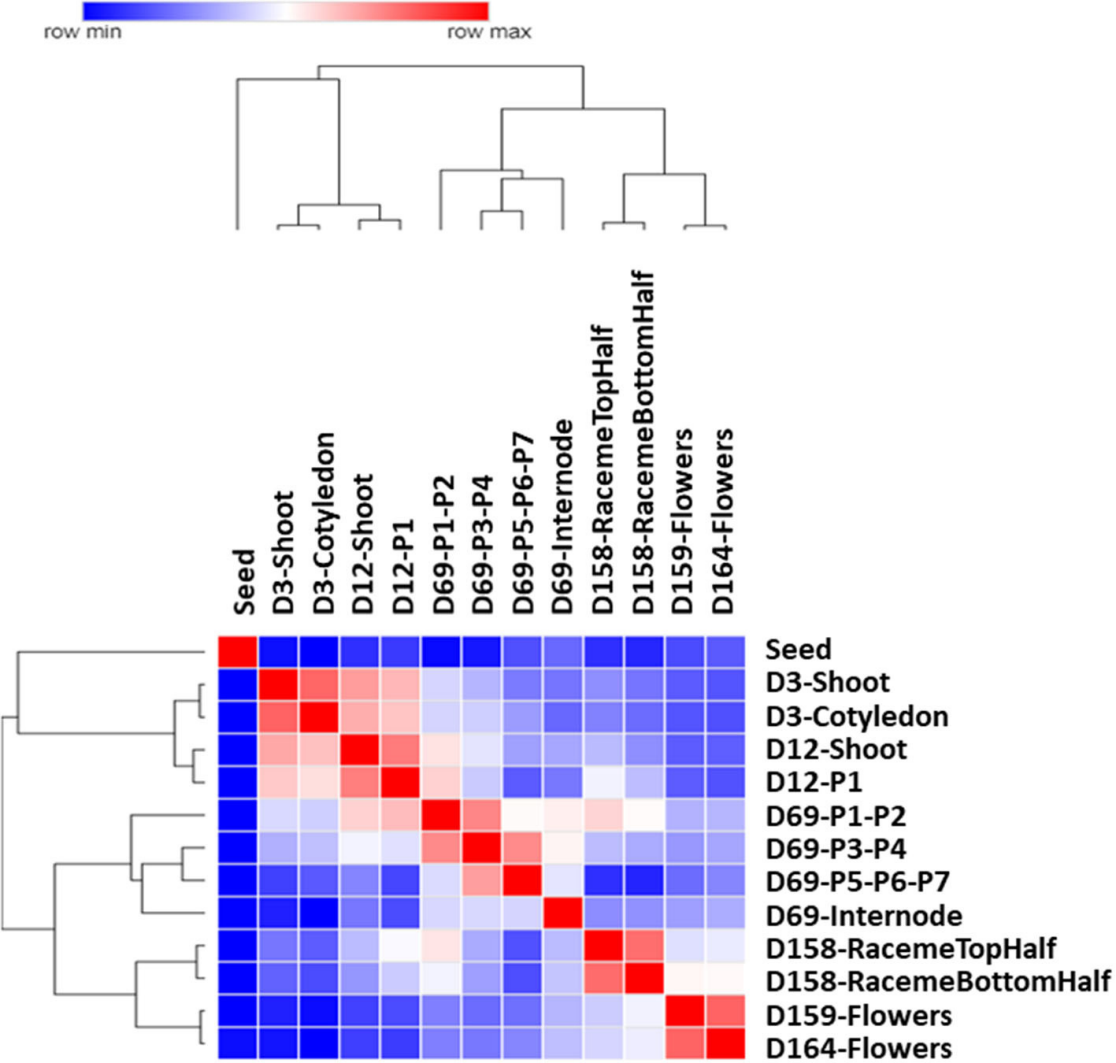
Gene expression patterns across different tissues of Chia. Heatmap of hierarchical clustering of the Pearson correlations for all 13 plant samples included in the gene expression atlas. Log2 transformed Fragments Per Kilobase of transcript per Million mapped reads (FPKM) values were used for generating the similarity matrix of transcripts. The color scale indicates the degree of correlation.

The hierarchical clustering of Pearson's correlations based upon the FPKM values of transcripts across all tissue samples provides an insight into the spatial and temporal gene expression pattern (Figure 2). Based on their plant structure and developmental stage attributes, the samples, for example, vegetative tissues, D3 (cotyledon and shoot), and D12 (shoot and very first leaf P1 at shoot apex), tend to be clustered together. D69 leaf samples from the developmental stages P1–P7 tend to be clustered together along with the D69 internode, which was located between the P6 and P7 nodes on the main stem. Similarly, samples of the reproductive plant structure of flowers (D159 and D164) and inflorescence (raceme top and bottom half) tend to be clustered together.

**Figure 3:**
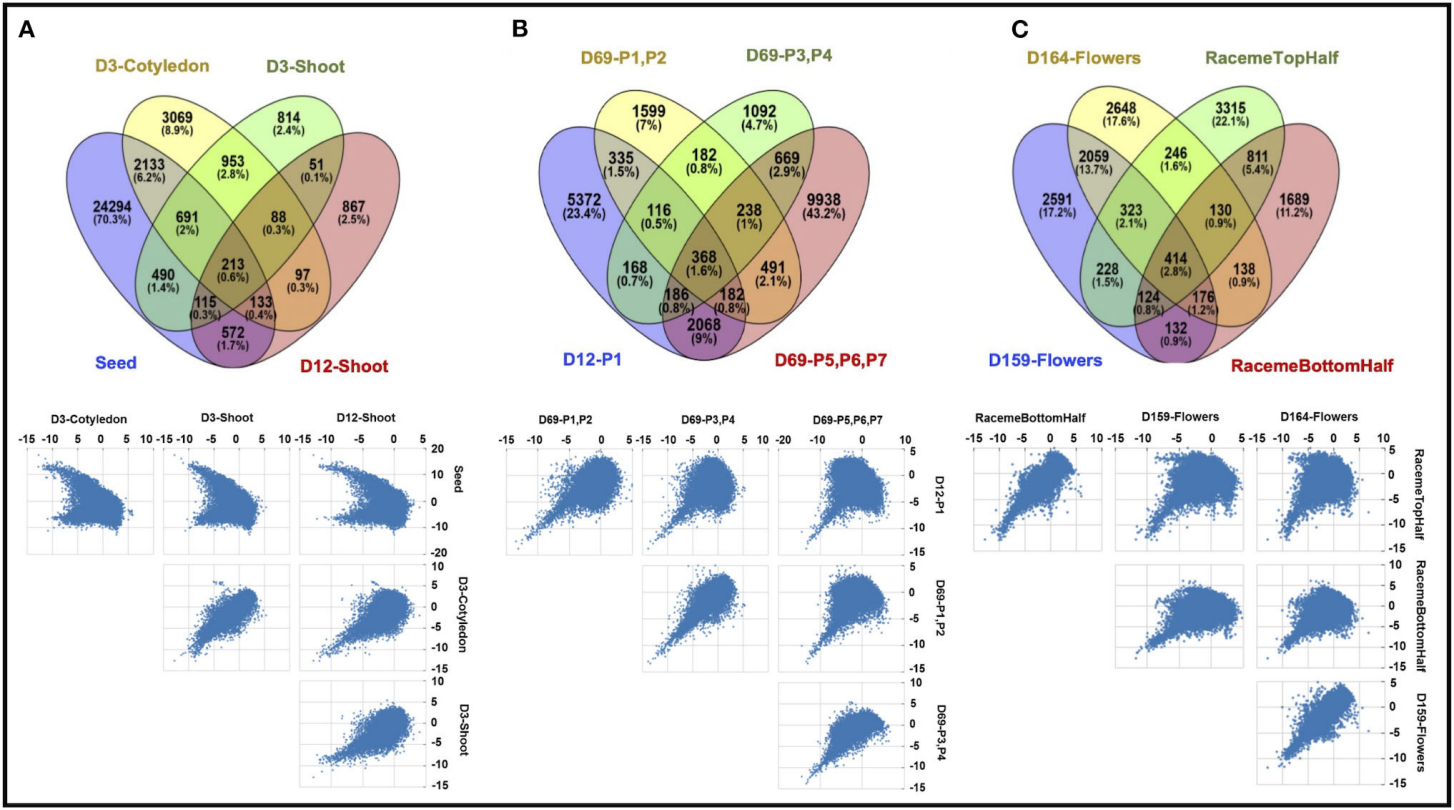
Comparison of differentially expressed transcripts (DETs) of Chia among **(A)** seed, D3-cotyledon, D3-shoot, and D12-shoot; **(B)** leaf samples from D12-P1 and D69-P1-P2, D69-P3-P4, D69-P5-P6-P7; **(C)** reproductive stage samples, including D158 inflorescence raceme top and bottom half, D159- and D164-flowers. The Venn diagrams in the upper panel represent common and unique DETs in each sample, and scatter plots in the lower panel represent the distribution pattern of DETs.

Overall, 53,200 of 82,385 transcripts showed a differential expression across the 13 samples; however, only 38,480 transcripts show a significant difference (log_2_ fold change ≥2) (see Supplementary Material 7). A summary of differentially expressed transcripts (DETs) across all tissues is shown in Table 2. In general, all tissue types show higher numbers of downregulated DETs compared to upregulated DETs except D3-cotyledon. Moreover, 1,696 DETs are common in all 13 samples, and a majority of DETs are regulated in more than one sample. Closely related tissue types show a maximum overlap in their transcriptome. Figure 3 shows common and unique DETs among the plant structure types that are grouped based on developmental stages. The samples collected from the initial growth stages (D3-cotyledon, D3-shoot, and D12-shoot) share only 213 DETs with the seeds, which have a remarkably distinct transcriptome (Figure 3A). Among all leaf developmental stages, only 368 DETs were common. Mature leaves (D69-P5-P6-P7) contain maximum DETs in both upregulated and downregulated categories (Figure 3B). Notably, in early leaf developmental stages (D12-P1 and D69-P1-P2), transcripts encoding for *Growth Regulating Factors* (*GRF2, GRF5)* and bHLH domain-containing (*SPEECHLESS*) transcription factors (TFs) were highly expressed. *GRF* TFs play an important role in leaf growth, and the bHLH *SPEECHLESS* factors are involved in stomata initiation and development (Kim et al., 2003; MacAlister et al., 2007; Kanaoka et al., 2008; Lampard et al., 2008). Among raceme inflorescence and flower tissues, 414 DETs were common and D164-Flowers had the maximum DETs (Figure 3C).

**Table 2:** Differentially expressed transcripts (DETs) across various developmental stages.

**Tissue type**	**Total DET**	**Tissue-specific**	**Upregulated (log_**2**_ FC ≥ 2)**	**Downregulated (log_**2**_ FC ≤ 2)**
Seed	28,641	13,450	6,284	13,429
D3-Cotyledon	7,377	1,781	2,632	1,746
D3-Shoot	3,415	495	970	1,161
D12-Shoot	2,136	270	52	1,521
D12-P1	8,795	2,038	770	3,976
D69-P1-P2	3,511	633	288	2,185
D69-P3-P4	3,019	556	479	1,701
D69-P5-P6-P7	14,140	3,504	1,884	6,637
D69-Internode	9,260	2,152	1,390	5,163
D158-RacemeTopHalf	5,591	1,183	770	2,865
D158-RacemeBottomHalf	3,614	804	852	1,860
D159-Flowers	6,047	1,136	1,274	2,883
D164-Flowers	6,134	879	969	3,353

Based on the expression profile, Chia transcripts can be grouped into 20 co-expression clusters (Supplementary Material 8). Notably, cluster 1 is enriched in DETs that show preferential expression in the seeds; cluster 5, 12, and 20 are enriched in DETs that show preferential expression in the vegetative tissues; and cluster 15 and 18 contain DETs showing preferential expression in reproductive tissues (Supplementary Material 9).

The 7,507 transcripts in cluster 1 showed preferential expression in the seeds, including the transcripts coding for the late embryogenesis abundant (LEA) proteins, seed storage proteins, oil body-associated proteins, and oleosin family proteins (Supplementary Material 7). In contrast, cluster 5 (4,616 transcripts) is enriched with the transcripts that show preferential expression in D3-cotyledon, D3-shoot including those coding for the TFs of basic leucine zipper family, photosystem I and II proteins, aquaporins, and calcineurin-like phosphoesterase domain-containing proteins (Supplementary Material 9).

Cluster 12 includes the 3,909 transcripts that show preferential expression in the D69-P5-P6-P7 leaf stages, including the transcripts coding for leucine-rich receptor-like kinases (LRR-RLKs) and wall-associated receptor kinases (*WAK*s), root hair-defective 3 GTP-binding (*RHD3*) domain-containing proteins, such as ABC, phosphate, and aluminum transporter proteins, cytochrome P450s, glycosyltransferases, and *WRKY* TFs (Supplementary Material 7). Both LRR-RLKs and WAKs are known to play roles in disease resistance and abiotic stress response (Harkenrider et al., 2016; Al-Bader et al., 2019; Wang et al., 2019; Amsbury, 2020; Zhang et al., 2020).

Cluster 20 consists of 2,173 transcripts that show preferential expression in the D69 Internode (Supplementary Material 9). It includes transcripts encoding MYB (*MYB54, MYB52*) and NAC TFs, xyloglucan endotransglucosylase, and receptor-like kinases (RLKs). *MYB54, MYB52*, and NAC TFs are known for their role in the internode development and secondary cell wall biosynthesis (Zhong et al., 2008; Grant et al., 2010; Cassan-Wang et al., 2013). Xyloglucan endotransglucosylase and RLKs are also involved in cell wall biosynthesis and expansion (Guo et al., 2009; Haruta et al., 2014).

Cluster 15 consists of 3,619 transcripts that showed a high expression in flowers. It includes the transcripts coding for beta-glucosidase, multidrug and toxic compound extrusion transporter proteins, cinnamyl alcohol dehydrogenase, germin-like proteins, pectin acylesterases, *MYB21* and *MYB24* TFs, *ZFP2* TF, GDSL lipases, and cytochrome P450s (Supplementary Material 9). *MYB21* and *MYB24* TFs play a role in petal, stamen, and gynoecium development in flowers (Reeves et al., 2012), and *ZFP2* controls floral organ abscission (Cai and Lashbrook, 2008). Cinnamyl alcohol dehydrogenases are involved in lignin biosynthesis in floral stem in *Arabidopsis* (Sibout et al., 2005), and germin-like proteins play an important role in response to pathogens (Zimmermann et al., 2006; Manosalva et al., 2009; Wang et al., 2013). Also, the 2,679 transcripts in cluster 18 showed an upregulation in the inflorescence tissues. Agamous-like MADS-box proteins and MYB family TFs that play a vital role in floral meristem development are enriched in cluster 18 (Supplementary Material 9). MYBs and MADS-box TFs are the essential regulators of various developmental processes (Zimmermann et al., 2004; Millar and Gubler, 2005; Yang et al., 2007; Gomez et al., 2011; Kobayashi et al., 2015). We find that transcripts annotated as terpene synthases show an upregulation in flowers as compared to the inflorescence tissues.

### Biological Pathways Enriched Across Different Development Stages

**Figure 4:**
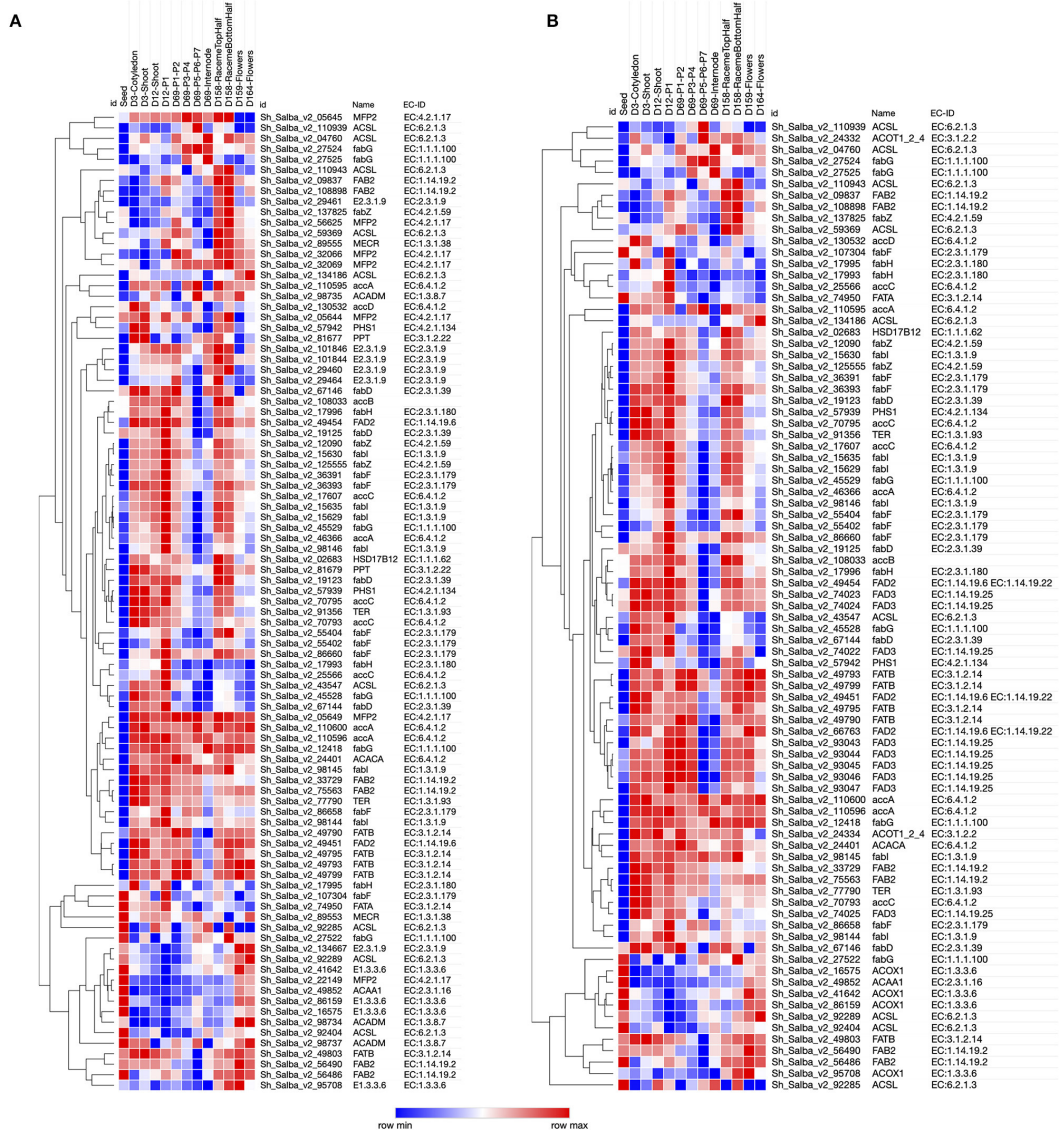
The plant structure and developmental stage-specific expression profile of transcripts involved in the fatty acid metabolism. **(A)** Fatty acid metabolism; **(B)** omega-3 (α-Linolenic acid) and omega-6 (Linoleic acid) unsaturated fatty acids metabolism.

The metabolic network representation across the developmental stages of Chia was determined by mapping to Kyoto Encyclopedia of Genes and Genomes (KEGG) pathways. We were successful in mapping a total of 5,555 Chia transcripts to 464 KEGG pathways, including starch and sucrose metabolism (PATH:ko00500), fatty acid metabolism (PATH:ko01040), phenylpropanoid biosynthesis (PATH:ko00940), photosynthesis (PATH:ko00195), fatty acid biosynthesis (PATH:ko00061), and amino acids metabolism (Supplementary Material 10). The expression pattern of transcripts encoding the enzymes for fatty acid metabolism and PUFA metabolism across different developmental stages was analyzed (Figure 4). Transcripts encoding acetyl-CoA carboxylase (EC 6.4.1.2), the very first enzyme catalyzing the conversion of acetyl-CoA to malonyl-CoA in the fatty acid biosynthesis, were highly expressed in all tissues except seeds. In the next reaction of this pathway, the malonyl group from malonyl-CoA is transferred to acyl carrier proteins (ACPs) for further elongation. We identified the transcripts for all the enzymes participating in the elongation steps. Acyl-ACP thioesterases (3.1.2.14) act in the last steps of fatty acid biosynthesis and serve as a determining factor for the generation of a variety of fatty acids within an organism. We further analyzed the expression of transcripts encoding for enzymes associated with PUFA metabolism (Figure 4B). We identified 32 fatty acid desaturase (FAD) transcripts from *FAD2, FAD3, FAD6, FAD7*, and *FAD8* families (Table 3). FADs are critical for catalyzing the fatty acid desaturation. Endoplasmic reticulum-localized *FAD2* and plastid-localized *FAD6* encode two ω-6 desaturases required to convert oleic acid to linoleic acid (18:2^Δ9, 12^) (Zhang et al., 2012). The desaturation of linoleic acid (18:2^Δ9, 12^) to α-linolenic acid (18:3^Δ9, 12, 15^) is catalyzed by the endoplasmic reticulum-localized FAD3 and plastid-localized FAD7 and FAD8 proteins (Dar et al., 2017; Xue et al., 2018).

**Table 3:** Transcripts annotated as Chia fatty acid desaturase (FAD).

**Fatty acid desaturases**	**Transcripts**
*FAD2*	Sh_Salba_v2_49454
	Sh_Salba_v2_49451
	Sh_Salba_v2_66763
*FAD3*	Sh_Salba_v2_74023
	Sh_Salba_v2_93044
	Sh_Salba_v2_93043
	Sh_Salba_v2_74025
	Sh_Salba_v2_93046
	Sh_Salba_v2_74024
	Sh_Salba_v2_74022
	Sh_Salba_v2_93047
	Sh_Salba_v2_93045
*FAD6*	Sh_Salba_v2_05727
	Sh_Salba_v2_05731
	Sh_Salba_v2_05730
	Sh_Salba_v2_05725
	Sh_Salba_v2_05728
	Sh_Salba_v2_05721
	Sh_Salba_v2_05724
*FAD7*	Sh_Salba_v2_69172
*FAD8*	Sh_Salba_v2_69173
	Sh_Salba_v2_90850
	Sh_Salba_v2_52578
	Sh_Salba_v2_69162
	Sh_Salba_v2_52570
	Sh_Salba_v2_52575
	Sh_Salba_v2_52576
	Sh_Salba_v2_69169
	Sh_Salba_v2_69166
	Sh_Salba_v2_52573
	Sh_Salba_v2_69174
	Sh_Salba_v2_69171

**Figure 5:**
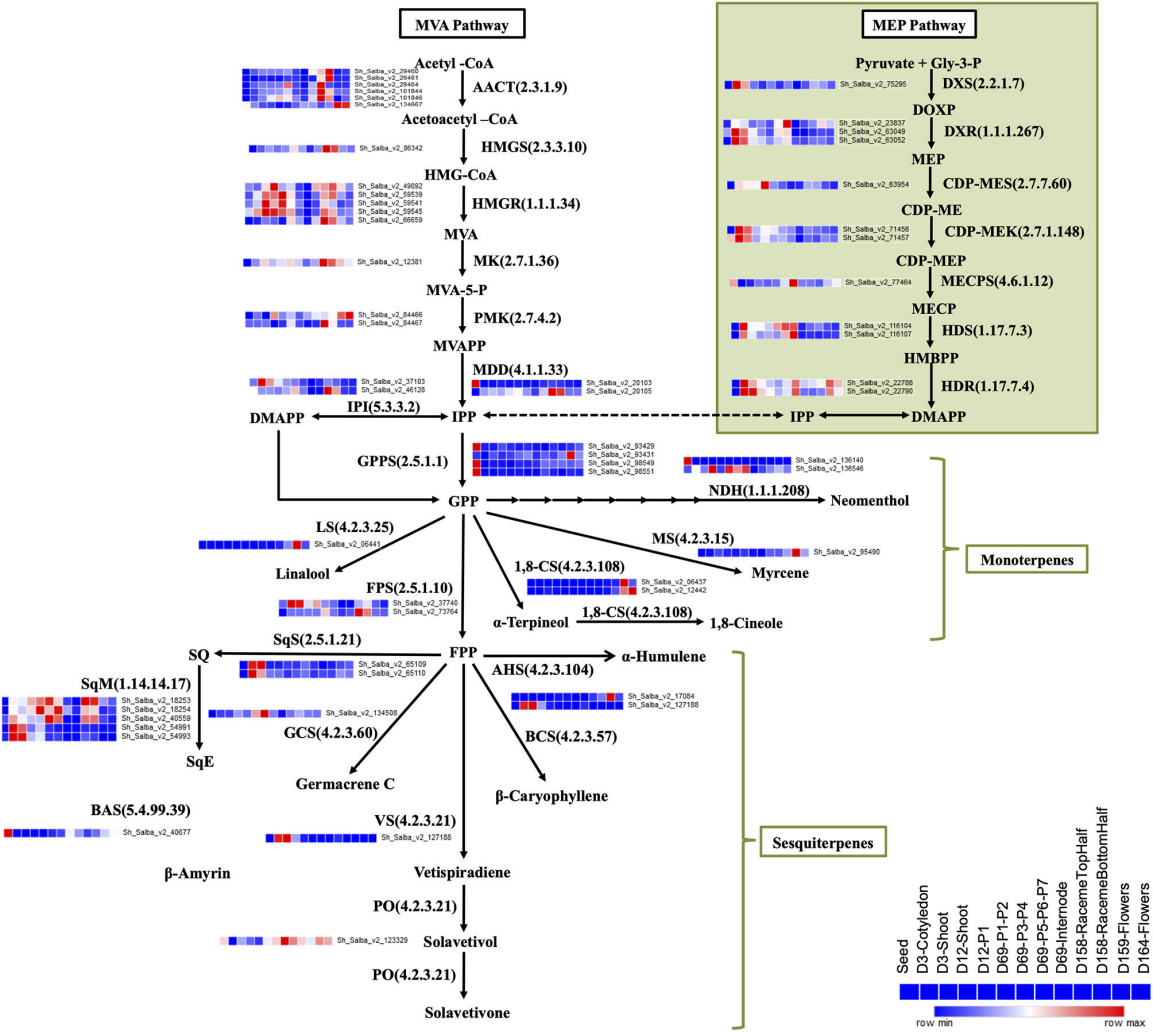
Tissue-specific expression of transcripts coding for enzymes of the terpenoid biosynthesis pathway. Biosynthesis of IPP, a central precursor for other terpenes biosynthesis, via cytosolic MVA and plastid-localized MEP pathways. Biosynthesis of various monoterpenes from GPP and sesquiterpenes from FPP. AACT, acetyl-CoA acetyltransferase; HMG-CoA, 3-hydroxy-3-methylglutaryl-CoA; MVA, mevalonate; MVA-5-P, mevalonate 5-phosphate; MVAPP, mevalonate diphosphate; IPP, isopentenyl diphosphate; DMAPP, dimethylallyl diphosphate; GPP, geranyl diphosphate; FPP, farnesyl diphosphate; HMGS, HMG synthase; HMGR, HMG reductase; MK, mevalonate kinase; PMK, phosphomevalonate kinase; MDD, Mevalonate diphosphosphate decarboxylase; IPI, IPP isomerase; GPPS, geranyl diphosphate synthase; FPPS, FPP synthase; Gly-3-P, glyceraldehyde-3-phosphate; DOXP, 1- deoxy-D-xylulose-5-phosphate; MEP, 2-C-methyl-D-erythritol-4-phosphate; CDP-ME, 4-diphosphocytidyl-2-C-methyl-D-erythritol; CDP-MEP, 4-diphosphocytidyl-2-C-methyl-D-erythritol-2-phosphate; MECP, C-methyl-D-erythritol-2,4-diphosphate; HMBPP, hydroxy methylbutenyl-4-diphosphate; DXS, DOXP synthase; DXR, DOXP reductoisomerase; CDP-MES, 2-C-methyl-D-erythritol4-phosphatecytidyl transferase; CDP-MEK, 4-(cytidine-5-diphospho)-2-C-methyl- D-erythritol kinase; MECPS, 2,4-C-methyl-D-erythritol cyclodiphosphate synthase; HDS, 1-hydroxy-2-methyl-2-(E)-butenyl-4-phosphatesynthase; HDR, 1-hydroxy-2-methyl-2-(E)-butenyl-4-phosphate reductase; NDH, neomenthol dehydrogenase; MS, myrcene synthase; 1,8-CS 1,8-cineole synthase; LS, linalool synthase; AHS, alpha-humulene synthase; BCS, beta-caryophyllene synthase; VS, vetispiradiene synthase; PO, premnaspirodiene oxygenase; SQ, squalene, SqS, squalene synthase; SqE, squalene epoxide; SqM, squalene monooxygenase; BAS, beta-amyrin synthase; GCS, germacrene C synthase.

Mint, a Lamiaceae family plant, is primarily known for the production of monoterpenes, e.g., menthol and limonene (Aharoni et al., 2005; Ahkami et al., 2015); however, the majority of Chia terpenes are sesqui-, di-, and tri-terpenes (Ma et al., 2012; Cui et al., 2015; Trikka et al., 2015). In our Chia dataset, we observed the expression profile of transcripts involved in the biosynthesis of terpenoid backbone, monoterpenes, and sesquiterpenes. Transcripts encoding the enzymes of the 2-C-methyl-D-erythritol 4-phosphate (MEP) and the mevalonate (MVA) pathways involved in the terpenoid backbone biosynthesis showed differential expression patterns among all tissue types (Figure 5). Transcripts for monoterpene synthases, such as 1,8-cineole synthase (EC 4.2.3.108), myrcene synthase (EC 4.2.3.15), and linalool synthase (EC 4.2.3.25), were highly expressed in the reproductive stage samples (Figure 5), indicating that flowers are the prime site for the biosynthesis of essential oils known to have therapeutic properties. However, transcripts for the sesquiterpene synthases, β-caryophyllene synthase (EC 4.2.3.57), α-humulene synthase (EC 4.2.3.104), germacrene synthase (EC 4.2.3.60), and solavetivone oxygenase (EC 4.2.3.21) were enriched in the vegetative tissues (Figure 5). Sesquiterpene, β-caryophyllene, α-humulene, Germacrene, and solavetivone are likely to play a role in herbivory defense.

### Identification of Distinct Tissue-Specific Networks of TFs in Chia

Transcription factors are the key regulators of a plant's growth and development. We identified 633 DETs encoding TFs belonging to 53 families (Supplementary Material 11) in our Chia transcriptome. The highest number of transcripts belongs to *MYB* (60), followed by *bHLH* (45), *NAC* (38), *bZIP* (32), *WRKY* (28), *C2H2* (27), *MYB-related* (25), *MADS-box* (26), *C3H* (24), *G2-like* (22), *Hd-ZIP* (22), *Trihelix* (17), *TCP* (14), *Dof* (13), *GATA* (13), *GRAS* (13), and *TALE* (13) gene families. The expression pattern of differentially expressed TFs across the developmental stages is shown in Supplementary Material 12A. To build a co-expression network, we first filtered out highly upregulated TFs in any of the 13 samples (≥5 log_2_ fold change) and then used 23 TFs as baits (nodes) and the FPKM value of 38,480 transcripts as an expression matrix. The seed-specific TF network showed 95,318 connections among 21 TFs and 4,629 transcript nodes (Supplementary Materials 12A, 13). A set of 92 transcripts (green) are connected to all 21 TFs. Two sets of 15 transcripts (teal) and 33 transcripts (pink) are connected to an ethylene responsive factor (ERF) (T112851) that is highly upregulated (log_2_ fold change 5.561) in the seeds. Most of the transcripts correlated to ERF (T112851) are downregulated in the seed and other tissue types. Two transcripts (T135081 and T106468) exclusively connected to NIN-like TF (T145088) are downregulated in the seeds and other tissues. A bZIP TF showed a connection to a transcript T120275, which is upregulated in the seeds but downregulated in other tissues. The MYB TF (T131530) is upregulated in the seeds and connected to a set of 34 transcripts (magenta) that were mostly downregulated in the seeds and other tissues. Bait transcripts are also correlated to each other, suggesting multiple regulatory modules within the network (Supplementary Material 12). Two TFs, *MYB* (T130985/Sh_Salba_v2_130985) and *C3H* (T121906/Sh_Salba_v2_121906), which were highly expressed in the D69-Internode but downregulated or absent in other tissues, showed connections (edges) to a set of 504 transcripts with no connection to any other bait nodes (Supplementary Material 12B).

### Identification of SSRs Markers

Simple sequence repeats are an important class of genetic markers widely used in molecular breeding applications. The identified SSRs from the transcriptome are highly advantageous as compared to the identified SSRs from the genome sequence. If an SSR from the transcribed region is polymorphic, it may have a direct impact on the expression, structure, stability of a transcript, and/or on a peptide sequence and the functional domains. We identified a total of 2,411 SSRs in the *de novo* assembled transcriptome represented by di-, tri-, and tetra-nucleotide motifs (Supplementary Material 14). The most abundant di-, tri-, and tetra-nucleotide motifs were CT (201), GAA (84), and AGTC (12), respectively (Supplementary Material 15). A total of 1,771 SSRs were present in the DETs, and 148 SSRs were found in the expressed transcripts mapped to at least one metabolic pathway (Supplementary Material 16).

## Discussion

To develop a repertoire of genetic and genomic data and knowledge resources on Chia, recent limited efforts have focused on the expression of lipid biosynthesis and terpene synthase genes in developing Chia seeds, roots, and leaves (Sreedhar et al., 2015; Peláez et al., 2019; Wimberley et al., 2020). We developed a comprehensive gene expression atlas for Chia from the 13 different plant structure types collected at various developmental stages using RNA-Seq coupled with the *de novo* transcriptome assembly approach (Table 1). This transcriptome atlas covers the complete assembly of about 86% of the genes present in the Chia genome. The assembled transcripts were annotated by using BLASTx and tBLASTx and translated into peptides using Transdecoder (v2.1.0); the peptides were annotated with GO terms and structurally conserved domains. Overall, the Chia transcriptome data set is comprehensive and covers the majority of genes participating in the cellular metabolic process, catalytic activity, regulation of gene expression, transport, ion binding, organelle development and function, and formation of macromolecular complexes. Thus, we present a much needed reference resource for the breeding and improvement of this important crop.

In the comparison of Chia transcripts datasets to genomic/transcriptome data sets from the six most closely related eudicots (Figure 1C), we found the topmost matching of a Chia transcript with the transcripts of perennial herbs, the red sage *S. miltiorrhiza* (Wenping et al., 2011) and the scarlet sage, *S. splendens* (Ge et al., 2014)—both species are rich in secondary metabolites and are used in traditional medicine. Furthermore, the Chia expression atlas provides insights into the functional relatedness of genes and their expression across the developmental stages and tissue types. Hierarchical clustering of Chia transcripts suggests the role of different gene families in the development of each growth stage, thus providing a foundation for studying the molecular mechanisms and metabolic pathways occurring in different tissues and developmental stages. For example, GRF family TFs, likely to play an essential role in regulating leaf number and size show high expression in D69-P1-P2 leaf stages. GRF family TFs play an essential role in the growth and development of leaf, and likewise express highly in D69-P1-P2 leaf stages. In *Arabidopsis, GRF1, GRF2*, and *GRF5* regulate leaf number and size (Kim et al., 2003; Horiguchi et al., 2005; Lee et al., 2009). Likewise, in mature leaves, transcripts coding for LRR-RLKs and WAKs proteins show high expression, which is consistent with their role in guard cells and stomatal patterning (Shpak et al., 2005) and biotic resistance (Harkenrider et al., 2016).

Essential oils, the secondary metabolites of the terpenoid biosynthesis pathways, are highly desired for their usage in medicine, food, and cosmetics, and have a potential survival benefit for the plant against insects, herbivores, and pathogens. In this Chia dataset, we identified transcripts encoding enzymes for the terpenoid backbone biosynthesis (Figure 5). Monoterpene synthases are involved in the biosynthesis of essential oils, and sesquiterpene synthases primarily contribute to the biosynthesis of insecticidal compounds. Chia flowers show a higher expression of monoterpene synthase transcripts (Figure 5) that code for 1, 8-cineole synthase (EC 4.2.3.108) and β-myrcene synthase (EC 4.2.3.15). Cineole and myrcene are found in fragrant plants and are known to have therapeutic medicinal properties such as sedative, anti-inflammatory, antispasmodic, and antioxidant (do Vale et al., 2002; Moss and Oliver, 2012; Bouajaj et al., 2013; Juergens, 2014; Khedher et al., 2017). The comparison of reproductive vs. vegetative tissue shows that monoterpene synthases were expressed highly in the reproductive tissues, and sesquiterpene synthases were prominent in the vegetative tissues. These findings confirm that flowers are involved in the synthesis of fragrant and essential oils whereas the vegetative tissues are rich in compounds, which are known for their herbivory defense and insecticidal properties. Phenylpropanoid and flavonoid biosynthesis pathways are also highly enriched in seeds and other tissue types (Supplementary Material 10).

Chia seeds are a rich source of PUFAs. We observed a lower expression of FAD transcripts in the seeds in comparison to other tissue types (Figure 4), suggesting that the seeds might serve as a storage organ for PUFAs rather than the synthesis site.

The correlation analysis hinted a significant relationship between highly upregulated TFs and the other DETs (Supplementary Material 12). The co-expression analysis suggested 21 TFs that are members of the B3, bZIP, ERF, WOX, AP2, MYB, C3H, EIL, LBD, DBB, NIN-like, and HSF families, play critical roles in the regulation of target gene expression across various developmental stages. We also observed that the two MYB and C3H zinc finger TFs were highly upregulated in the D69-Internode (Supplementary Material 12). Their expression is consistent with the reports on their orthologs' role in internode elongation and development processes (Zhong et al., 2008; Kebrom et al., 2017; Gómez-Ariza et al., 2019). Of the 21 TFs highly expressed in the seed samples, T112851 is an AP2/ERF family member. Its homologs are known to play a role in dehydration-induced response as the DREB2A proteins that are involved in response to drought, salt, and low-temperature stress (Nakashima et al., 2000; Sakuma et al., 2002). We expected to see such stress-responsive genes in seed samples because the seeds undergoes dehydration during their maturation (Naithani et al., 2017).

The conventional use of the BUSCO gene analysis is to assess the completeness of genome and transcriptome. However, in the first report of its kind known to us, we used the Chia BUSCO gene set to assess the gene expression data quality. A baseline transcript abundance or the expression levels of a conserved Viridiplantae BUSCO gene set were compared between Chia and *Arabidopsis* (Supplementary Materials 17, 18). In the Chia transcriptome atlas data set, 601 transcripts were mapped to 411 BUSCO genes. They showed sample-specific transcript abundance: 49 Chia transcripts and their *Arabidopsis* homologs show a similar higher abundance in dry seeds, and 28 transcripts in the D69 leaves and their *Arabidopsis* homologs show similar transcript abundance profiles in leaf and plant parts carrying leaf-like structures. The conserved expression profiles of homologous BUSCO genes in taxonomically diverse Chia and *Arabidopsis* plants (Supplementary Material 17) support the high-quality of Chia transcriptome generated in this study. Our gene expression validation analysis carries more genes (>400), uses the conserved green plant BUSCO gene set, and improves the typical use of the reverse transcription (RT)-PCR method to quantify 5–10 genes for validating transcript abundance. This analysis was only possible due to the availability of high-quality publicly available transcriptome data sets provided by the EMBL-EBI Gene Expression Atlas (https://www.ebi.ac.uk/gxa/home) (Papatheodorou et al., 2018), supported by the Ensembl Plants (http://plants.ensembl.org/index.html) (Howe et al., 2020) and the Gramene databases (http://www.gramene.org) (Tello-Ruiz et al., 2021).

Further analysis of the *de novo* assembled Chia transcriptome revealed 2,411 SSRs (Supplementary Material 15). SSRs are an important class of genetic markers widely used in molecular breeding applications. The identified SSRs from the transcriptome are highly advantageous as compared to the identified SSRs from the genome because they are from the transcribed region, and if they are polymorphic, they may have a direct impact on the expression, structure, and stability, of the transcript and the peptide (Supplementary Material 16). The SSRs identified in the Chia reference transcriptome are a valuable resource for breeding and genetic improvement of the crop.

Overall, this is the first study on the generation of a well-annotated plant structure-specific reference transcriptome atlas for Chia, a neo model, and an agronomically important crop. We expect that the raw and analyzed Chia transcriptome sequence data and 2,411 SSRs identified in this study would serve as an important resource for the researchers working on Chia and other important plant species of the mint family. The transcriptome data will greatly help in correcting errors in the future genome assembly of *S. hispanica*, the identification of gene models, improving the gene and genome annotation, and the development of a Chia-specific metabolic network. This transcriptome study is expected to initiate opportunities to undertake comparative and functional genomics, pathway analyses, and genome to phenome studies in Chia.

## Materials and Methods

### Plant Material, Growth Conditions, and Sampling

Seeds of Chia (*S. hispanica* L.) were bought online from Ancient Naturals, LLC, Salba Corp., N.A., were sown in autoclaved soils, and watered thoroughly under controlled greenhouse conditions. All seeds germinated on the third day after sowing (DAS). Since the primary seed material was expected to be a heterogeneous mixture, biological replicates for each tissue type were collected from three randomly chosen plants. The description of the samples collected from various developmental stages and tissue types is shown in Table 1. The tissue samples include seeds, green cotyledons, shoots after 3 and 12 DAS, leaves from day-12 (D12-P1) and day-69 DAS (D69-P1-P7), an internode (D69-Internode) between the P5 and P6 leaf nodes collected on day-69 DAS, top and bottom halves of the raceme inflorescence from 158 DAS (D158) carrying pre-anthesis flowers, and flowers from the day-1 and day-5 of flowering (anthesis stage) (D159) and 164 (D164) DAS. Collected samples were immediately frozen in liquid nitrogen and stored at −80°C.

### Sample Preparation and Sequencing

Total RNA from the frozen tissues was extracted as per the manufacturer's protocol using the RNA Plant reagent (Invitrogen Inc., Waltham, MA, USA), RNeasy kits (Qiagen Inc., Germantown MD, USA), and treatment with RNase-free DNase (Life Technologies Inc., Carlsbad, CA, USA). Total RNA concentration and quality were determined by using ND-1000 spectrophotometer (Thermo Fisher Scientific Inc., Waltham, MA, USA) and Bioanalyzer 2100 (Agilent Technologies Inc., Santa Clara, CA, USA). Samples were prepared separately from each of the three biological replicates of each tissue type by using the TruSeqTM RNA Sample Preparation Kits (v2) and sequenced by using the Illumina HiSeq 2500 instrument (Illumina Inc., San Diego, CA, USA) at the Center for Genomic Research and Biocomputing, Oregon State University, Corvallis, OR, USA.

### *De novo* Transcriptome Assembly and Quality Estimation

FASTQ file generation from the RNA-Seq sequences was done by using the CASAVA software v1.8.2 (Illumina Inc., San Diego, CA, USA). Read quality was assessed by using FastQC, and poor-quality reads were removed with Sickle v. 1.33 (–q = 20) (“najoshi/sickle”). The transcripts were assembled by using Velvet (v1.2.10), which uses De Bruijn graphs to assemble short reads (Zerbino and Birney, 2008). An assembly of 67 and 71 k-mer lengths was performed by using all tissue-specific reads. The assemblies produced by Velvet were merged into a single consensus assembly by Oases (v0.2.08) (Schulz et al., 2012), which produced transcript isoforms using read sequence and pairing information. Quality estimation for reducing redundancy in a transcript assembly (a quality control check for *de novo* assembled transcriptome) was carried out by using CD-HIT-EST (Li and Godzik, 2006), TransRate (Smith-Unna et al., 2016), and QUAST (Gurevich et al., 2013) software packages. The assembled transcripts passing the CD-HIT-EST quality control step were used for further downstream analyses and considered a reference transcriptome for differential gene expression analyses. The BUSCO analysis (Simão et al., 2015) on the Chia transcriptome was also performed at the Galaxy platform (Afgan et al., 2018) to assess the completeness of the transcriptome set and coverage of the Chia gene set. We used the BUSCO version 4.1.2 and the Viridiplantae (green plant) lineage-specific data set viridiplantae_odb10, with a 425 reference single-copy core gene set.

### Functional Annotation and Pathway Enrichment Analysis

Assembled transcripts were annotated by using BLASTx and tBLASTx with a value of *E* cutoff of 10^−10^. The assembled transcripts were translated into peptides by using Transdecoder (v2.1.0) [“TransDecoder (Find Coding Regions Within Transcripts)”] with a minimum peptide length of 50 or more amino acids. Transdecoder used the BLASTp and PfamA search results to predict the translated ORF. The resulting peptides were analyzed by using the InterProScan Sequence Search (v5.17.56) (Jones et al., 2014) hosted by the Discovery Environment and powered by CyVerse (Joyce et al., 2017). We used the agriGO Analysis Toolkit (Tian et al., 2017) to identify statistically enriched function groups of transcripts. agriGO uses a Fisher's exact test with a Yekutieli correction for the false discovery rate calculation. Significance cutoffs were set at a value of *p* equal to 0.05 and a minimum of five mapping entries per GO term. KAAS-KEGG automation server was used for an ortholog assignment and a pathway analysis (Moriya et al., 2007).

### Gene Expression and Clustering

Bowtie2 (Langmead and Salzberg, 2012) was used to align the sequence reads from each tissue type to the assembled transcriptome. The RSEM software package (Li and Dewey, 2011) was used to estimate the transcript expression counts (FPKM) from the aligned sequence reads. The count data obtained from RSEM was used in EBSeq (Leng et al., 2013) to identify differentially expressed genes based on the false discovery rate corrected value of *p* is 0.05. Heatmaps were generated by using Morpheus (https://software.broadinstitute.org/morpheus) and MEV (version 4.8.1) (MEV: MultiExperiment Viewer, 2017) to cluster the expression data from Chia. Log_2_ transformed fold change value for each transcript was used as an input (value of p being 0.1). Due to the orders of magnitude in the expression of transcripts between the tissue types, we chose several data normalization methods for cluster generation. Unit variance, median centering of transcripts, and the summation of squares were applied to the dataset. In the investigation of individual gene families, transcripts were hierarchically clustered using a Pearson correlation. The Chia transcripts were mapped to the BUSCO genes, and their *Arabidopsis* homologs were compared by using the *Arabidopsis* dataset E-MTAB-7978 (Mergner et al., 2020) from the EMBL-EBI Gene Expression Atlas (https://www.ebi.ac.uk/arrayexpress/experiments/E-MTAB-7978).

### Co-expression and Network Analysis

The TF transcripts were classified based on homology searches in Plant TFDB database v5.0 (http://planttfdb.cbi.pku.edu.cn) (Jin et al., 2017) and BlastX searches against *A. thaliana*. For the co-expression analysis, the CoExpNetViz tool (Tzfadia et al., 2015) was used. This tool utilizes a set of query or bait genes as an input and a gene expression dataset. TF transcripts displaying a maximum expression cutoff of log_2_ transformed FPKM ≥ 5 were used as baits, and DETs displaying a maximum expression cutoff of log_2_ transformed FPKM ≥ 2 were used as an expression matrix. Baits and an expression matrix were loaded in the CoExpNetViz tool, and the analysis was run on default parameters to calculate co-expression with the setting of the Pearson correlation coefficient. For an expression matrix, the transcripts are considered as co-expressed if their correlation does not lie between the lower (1st) and upper (99th) percentile of the distribution of correlations between a sample of genes per gene expression matrix. The output files from the CoExpNetViz tool were used for displaying a gene-co-expression network by using Cytoscape (version 3.7.1).

### Identification of SSRs

Multiple-length nucleotide SSRs were identified in the transcripts of the CD-HIT-EST assembly by using the stand-alone version of the SSR Identification Tool (SSRIT) (Temnykh, 2001).

## Data Availability Statement

The raw sequencing data from all cDNA libraries were deposited at EMBL-EBI ArrayExpress under the experiment number E-MTAB-5515 (https://www.ebi.ac.uk/arrayexpress/experiments/E-MTAB-5515). All the analyzed data for this project is accessible from the Chia Genomics Database (ChiaGDB: http://salvia.cgrb.oregonstate.edu/) and the Pankaj Jaiswal (2017). Comparative analysis of reference transcriptome atlas and insight into essential fatty acids and terpenoid biosynthesis pathways from Chia (Salvia hispanica). CyVerse Data Commons. DOI 10.7946/P2192W.

## Author Contributions

PJ and MG conceptualized the project. EH and JP helped MG in determining growth conditions, maintaining plants, sample collection, and extracting RNA. PG, MG, and SN did the data analysis. PG, MG, SN, and PJ wrote the manuscript. All authors reviewed and approved the manuscript.

## Conflict of Interest

The authors declare that the research was conducted in the absence of any commercial or financial relationships that could be construed as a potential conflict of interest.

## Supplementary Material

Supplementary Material 1 A summary of the raw and clean reads obtained after the sequencing and preprocessing, respectively, and reads aligned to the reference transcriptome.

Supplementary Material 2 Quality assessment of merged (column 2), CD-HIT-EST (column 3), and TransRate (column 4) assemblies using QUAST.

Supplementary Material 3 Workflow of Chia transcriptome sequencing and downstream analysis.

Supplementary Material 4 Benchmarking Universal Single-Copy Orthologs (BUSCO) analysis of assembled Chia transcriptome.

Supplementary Material 5 Functional annotation of Chia peptides using InterProScan.

Supplementary Material 6 Gene ontology (GO) annotations of Chia peptides.

Supplementary Material 7 Tissue-specific transcripts and their differential expression data for all Chia transcripts.

Supplementary Material 8 *k*-means clustering of transcripts depicting tissue-specific gene expression across different developmental stages. The *y*-axis in each cluster denotes the mean-centered log_2_ transformed FPKM values ranging from +17 to −17.

Supplementary Material 9 Chia transcripts clustered in 20 clusters.

Supplementary Material 10 Transcripts mapped to Kyoto Encyclopedia of Genes and Genomes (KEGG) pathways.

Supplementary Material 11 Differentially expressed transcription factors (TFs) across various developmental stages.

Supplementary Material 12 Seed- and internode-specific transcription network. **(A)** The seed-specific TF network shows connections among 21 TFs and 4,629 transcripts (targets) and **(B)** the internode-specific TFs network shows connections among 2 TFs and 504 transcript (targets).

Supplementary Material 13 Transcription network in seed and internode.

Supplementary Material 14 Frequency distribution of simple sequence repeats (SSRs) in Chia transcripts.

Supplementary Material 15 SSR motifs in Chia transcripts.

Supplementary Material 16 SSRs identified in transcripts coding for enzymes.

Supplementary Material 17 Heat maps of transcript abundance (log2 FPKM) for **(A)** all BUSCO transcripts in Chia, **(B)** a comparison of seed-enriched Chia BUSCO transcripts and their *Arabidopsis* homologs, and **(C)** a comparison of leaf-enriched Chia BUSCO transcripts and their *Arabidopsis* homologs.

Supplementary Material 18 FPKM values of Chia BUSCO transcripts across different developmental stages and their *Arabidopsis* homologs.

## Abbreviations

DAF: Days after flowering
DAS: Days after sowing.
