# Supplementary figures and images for: Chia (Salvia hispanica) Gene Expression Atlas Elucidates Dynamic Spatio-Temporal Changes Associated With Plant Growth and Development
Source: Front Plant Sci. 2021 Jul 20;12:667678. doi: 10.3389/fpls.2021.667678 (PMC8330693; doi:10.3389/fpls.2021.667678)

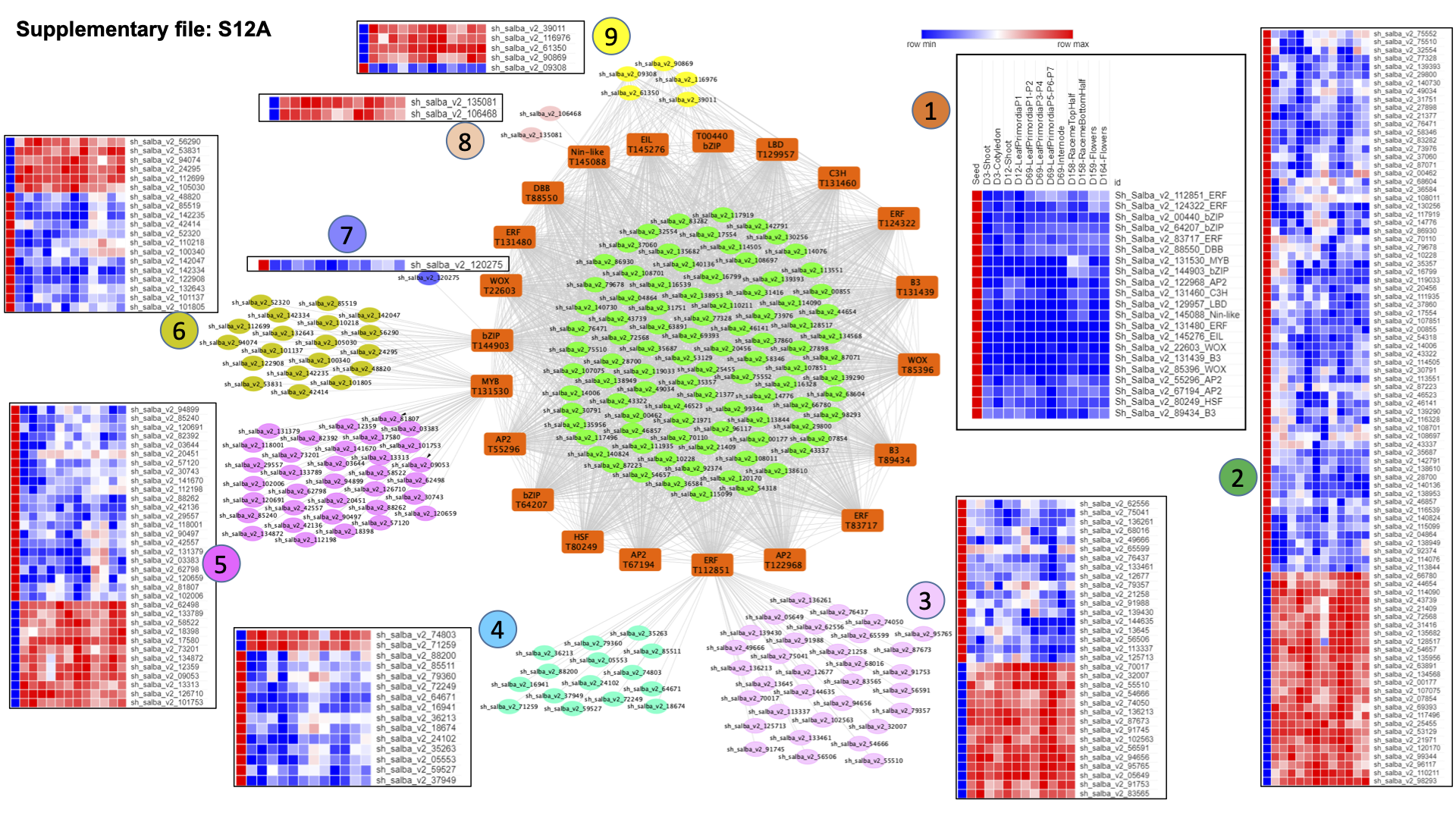

Supplement: Supplementary Material 1 — A summary of the raw and clean reads obtained after the sequencing and preprocessing, respectively, and reads aligned to the reference transcriptome. [file Data_Sheet_1.ZIP › Supplementary file S12.tiff]

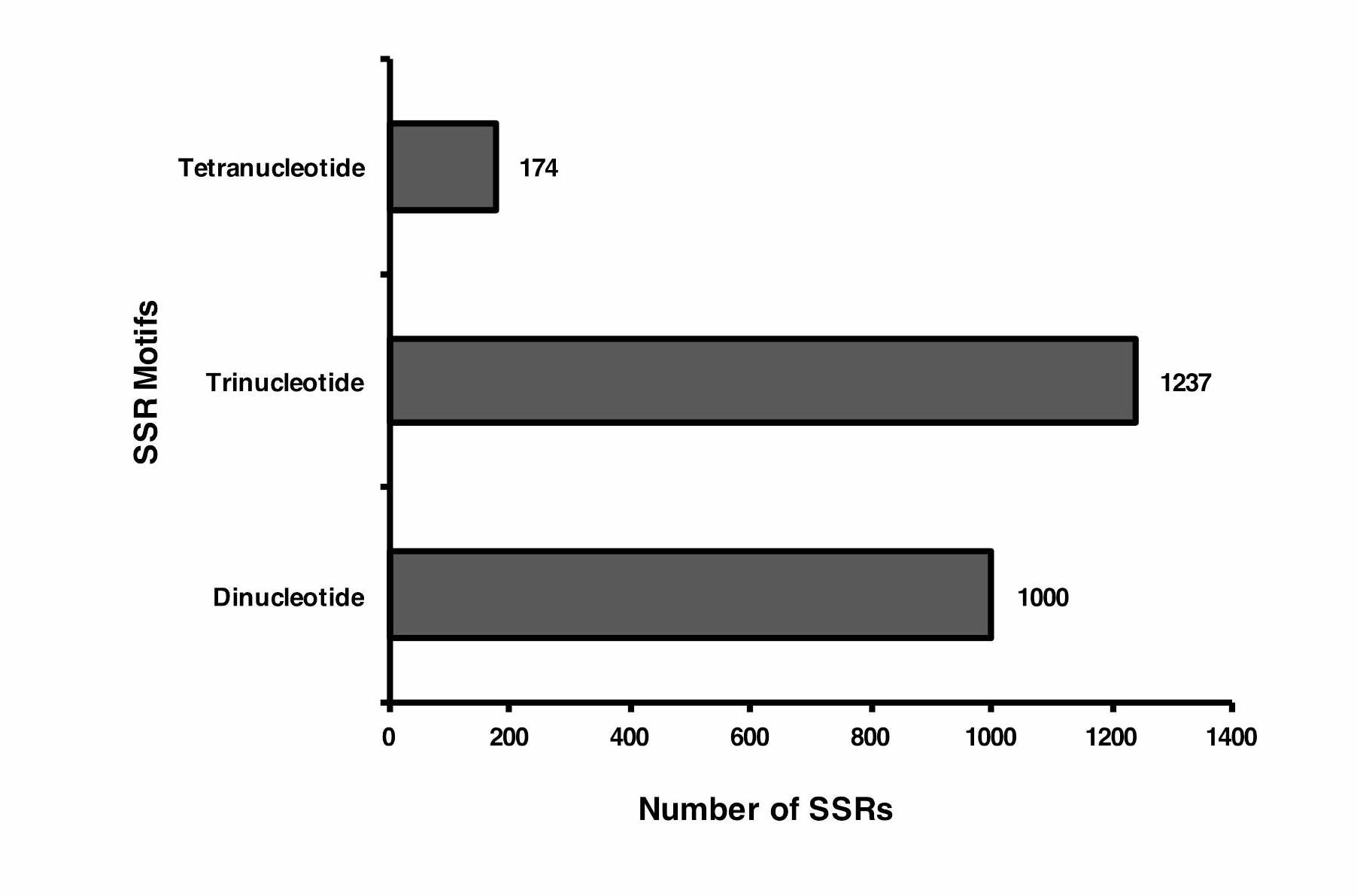

Supplement: Supplementary Material 1 — A summary of the raw and clean reads obtained after the sequencing and preprocessing, respectively, and reads aligned to the reference transcriptome. [file Data_Sheet_1.ZIP › Supplementary file S14.jpg]

Supplementary file S17

A

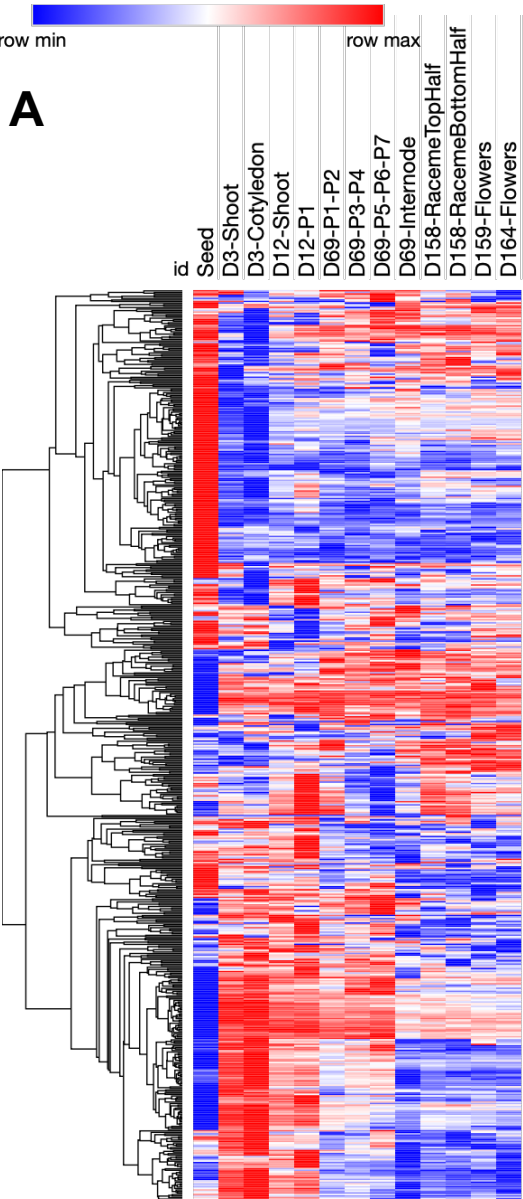

B

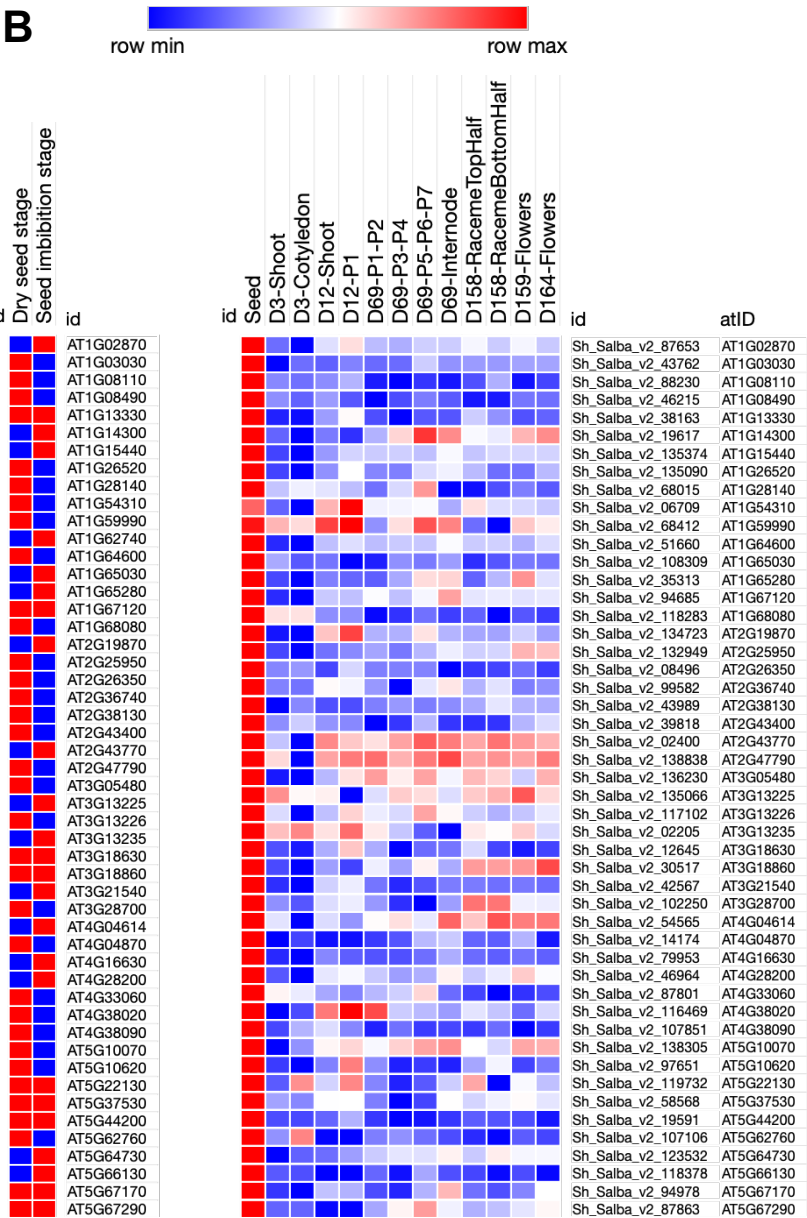

C

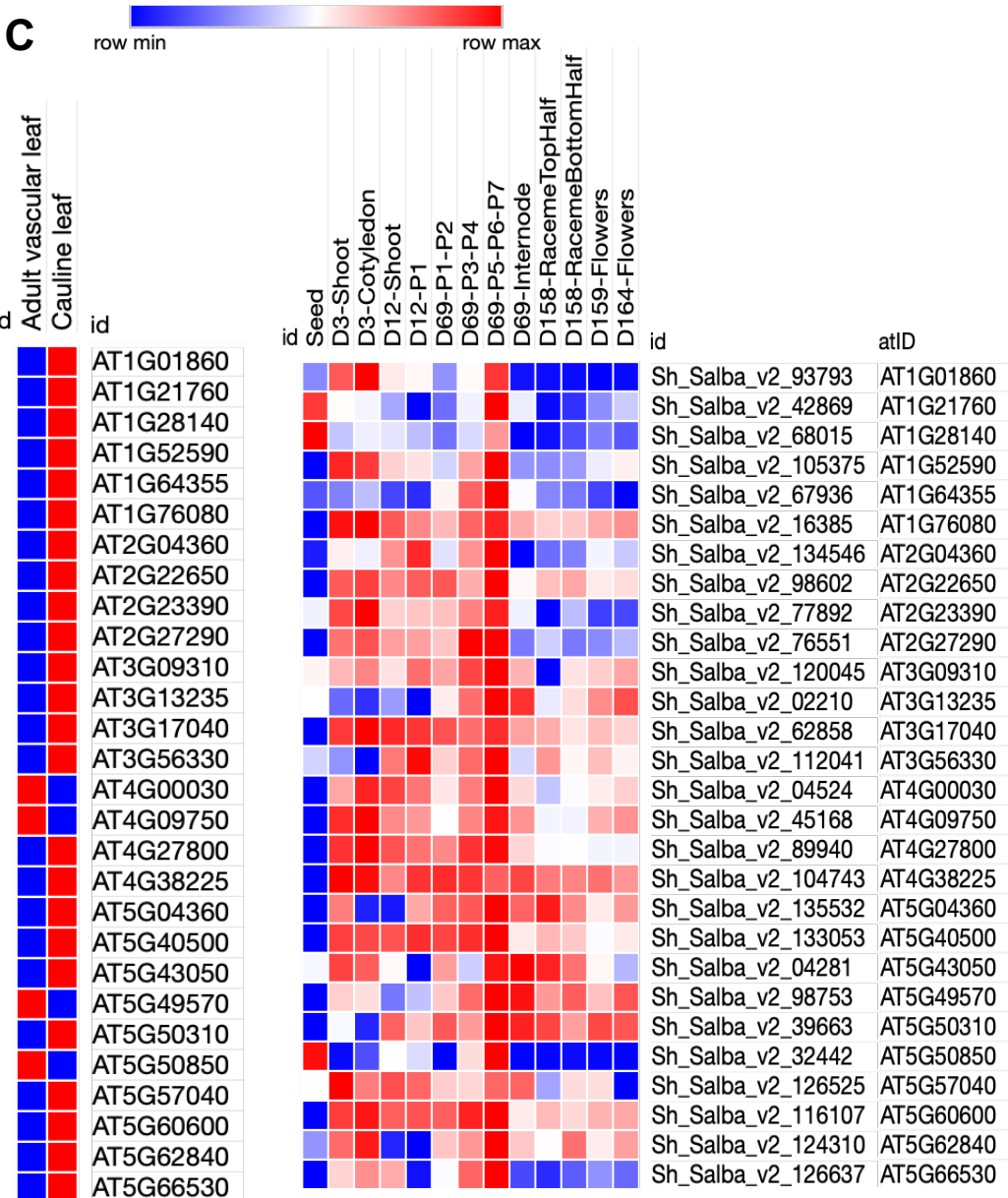

Supplement: Supplementary Material 1 — A summary of the raw and clean reads obtained after the sequencing and preprocessing, respectively, and reads aligned to the reference transcriptome. [file Data_Sheet_1.ZIP › Supplementary file S17.pdf]

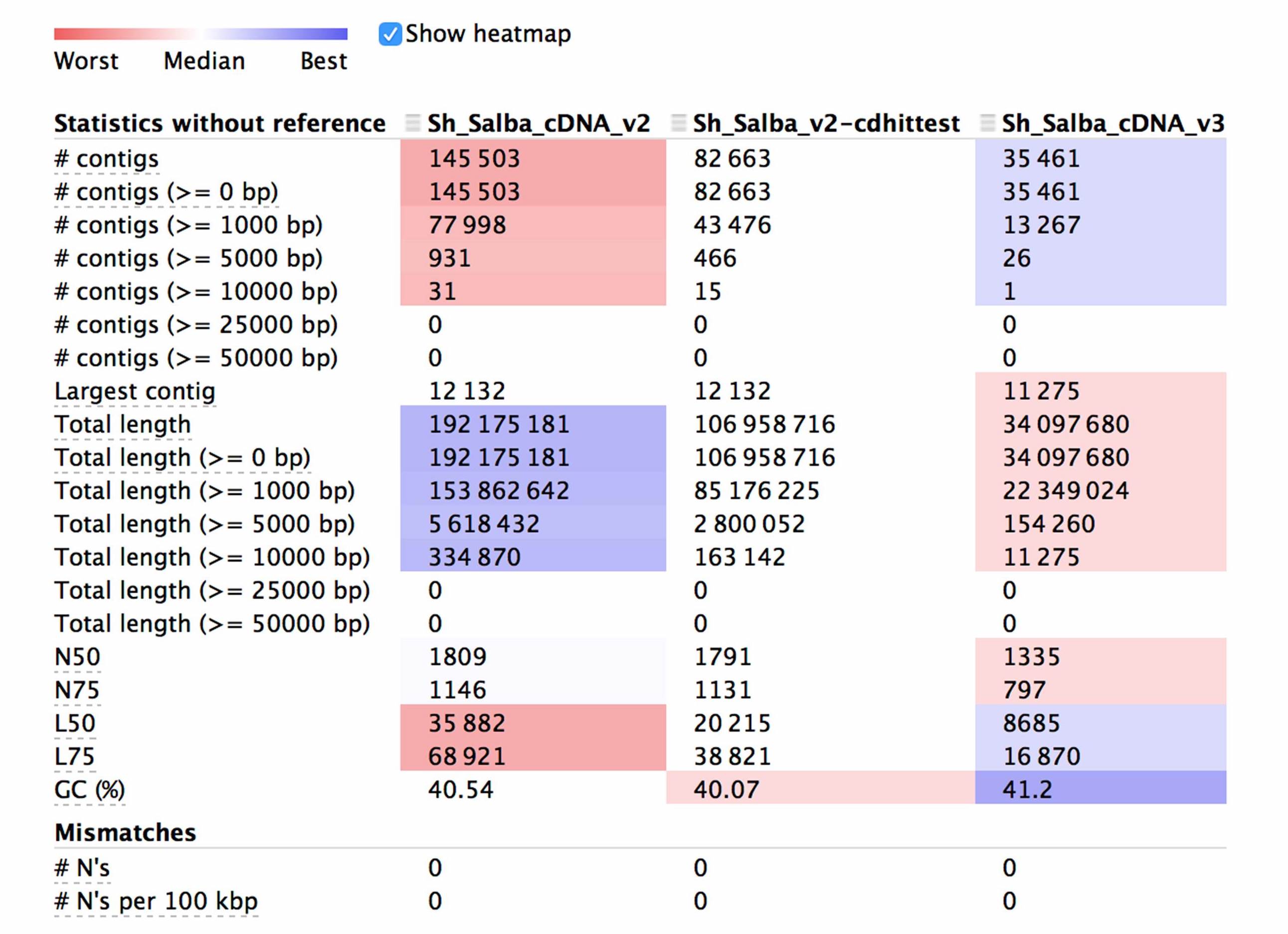

Supplement: Supplementary Material 1 — A summary of the raw and clean reads obtained after the sequencing and preprocessing, respectively, and reads aligned to the reference transcriptome. [file Data_Sheet_1.ZIP › Supplementary file S2.jpg]

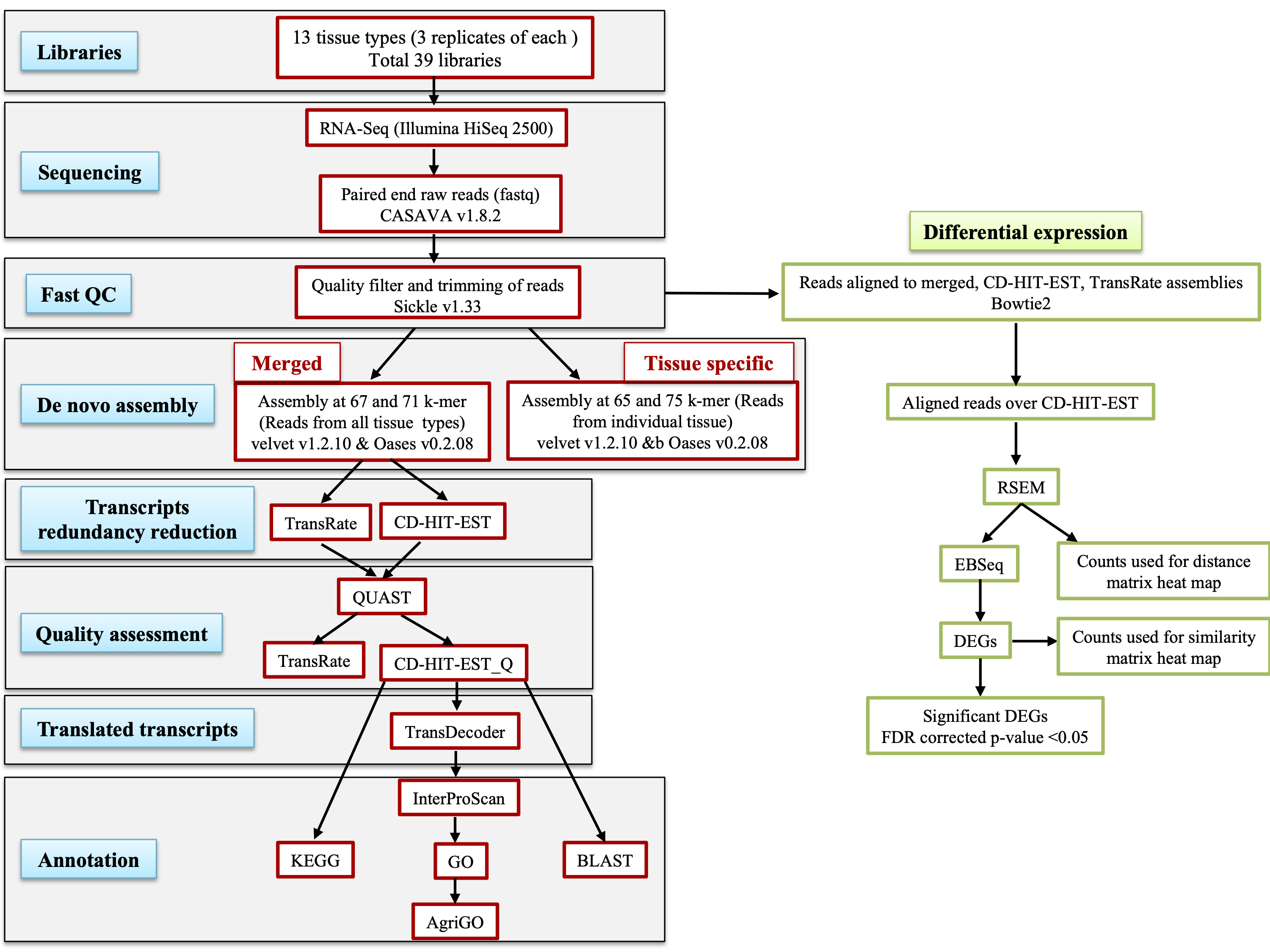

Supplement: Supplementary Material 1 — A summary of the raw and clean reads obtained after the sequencing and preprocessing, respectively, and reads aligned to the reference transcriptome. [file Data_Sheet_1.ZIP › Supplementary file S3.tiff]

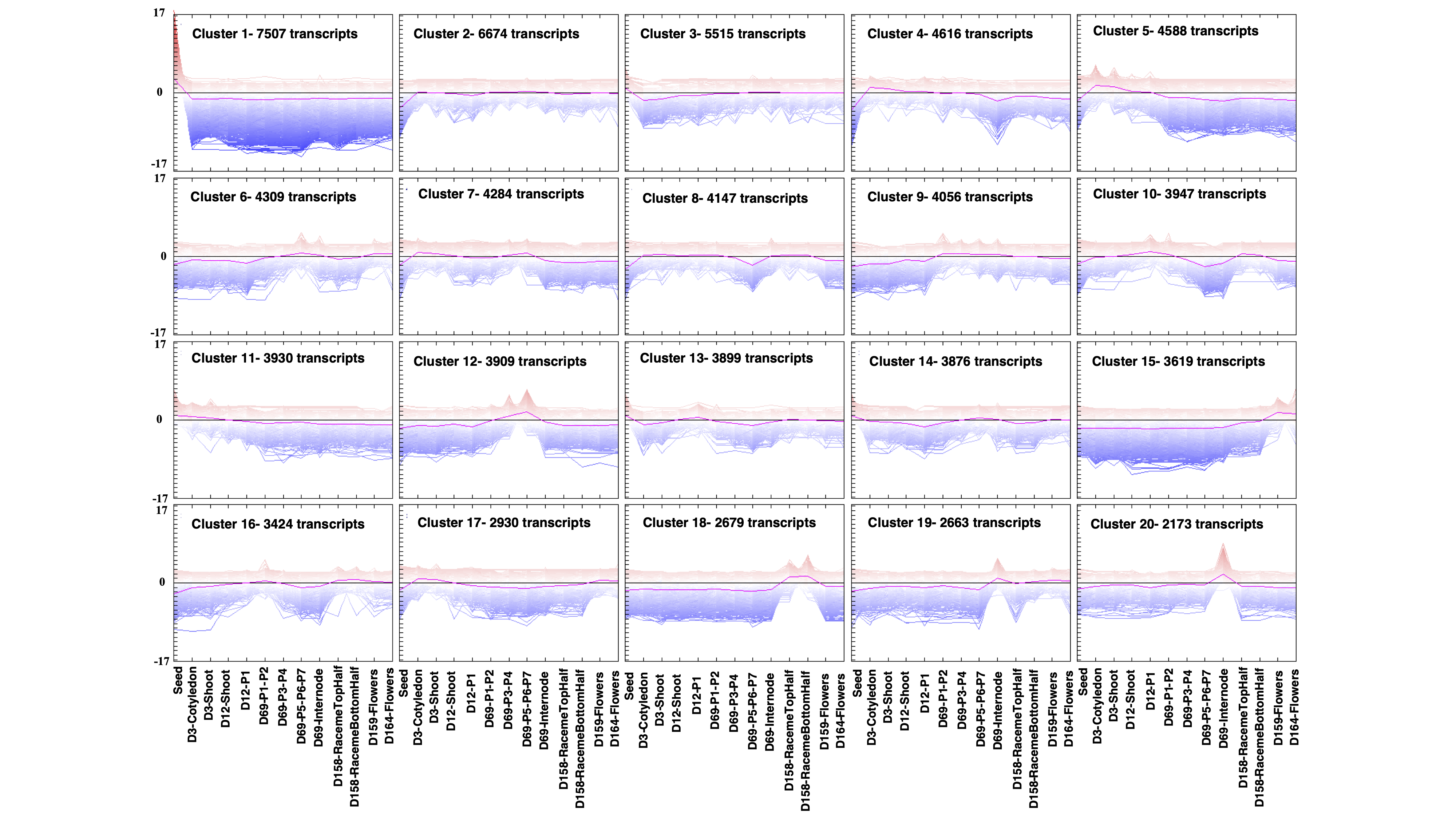

Supplement: Supplementary Material 1 — A summary of the raw and clean reads obtained after the sequencing and preprocessing, respectively, and reads aligned to the reference transcriptome. [file Data_Sheet_1.ZIP › Supplmentary file S8.tiff]
